# Notch4 mediates vascular remodeling via ERK/JNK/P38 MAPK signaling pathways in hypoxic pulmonary hypertension

**DOI:** 10.1186/s12931-022-01927-9

**Published:** 2022-01-11

**Authors:** Mingzhou Guo, Mengzhe Zhang, Xiaopei Cao, Xiaoyu Fang, Ke Li, Lu Qin, Yuanzhou He, Jianping Zhao, Yongjian Xu, Xiansheng Liu, Xiaochen Li

**Affiliations:** 1 Department of Pulmonary and Critical Care Medicine, Tongji Hospital, Tongji Medical College, Huazhong University of Science and Technology, 1095 Jiefang Avenue, Wuhan, 430030 China; 2 Key Laboratory of Respiratory Diseases, National Ministry of Health of the People’s Republic of China and National Clinical Research Center for Respiratory Disease, Wuhan, China; 3 Department of Pediatrics, Tongji Hospital, Tongji Medical College, Huazhong University of Science and Technology, Wuhan, China

**Keywords:** Hypoxic pulmonary hypertension, Human pulmonary arterial smooth muscle cells, Notch4, Mitogen-activated protein kinase

## Abstract

**Background:**

Hypoxic pulmonary hypertension (HPH) is a chronic progressive advanced disorder pathologically characterized by pulmonary vascular remodeling. Notch4 as a cell surface receptor is critical for vascular development. However, little is known about the role and mechanism of Notch4 in the development of hypoxic vascular remodeling.

**Methods:**

Lung tissue samples were collected to detect the expression of Notch4 from patients with HPH and matched controls. Human pulmonary artery smooth muscle cells (HPASMCs) were cultured in hypoxic and normoxic conditions. Real-time quantitative PCR and western blotting were used to examine the mRNA and protein levels of Notch4. HPASMCs were transfected with small interference RNA (siRNA) against Notch4 or Notch4 overexpression plasmid, respectively. Cell viability, cell proliferation, apoptosis, and migration were assessed using Cell Counting Kit-8, Edu, Annexin-V/PI, and Transwell assay. The interaction between Notch4 and ERK, JNK, P38 MAPK were analyzed by co-immunoprecipitation. Adeno-associated virus 1-mediated siRNA against Notch4 (AAV1-si-Notch4) was injected into the airways of hypoxic rats. Right ventricular systolic pressure (RVSP), right ventricular hypertrophy and pulmonary vascular remodeling were evaluated.

**Results:**

In this study, we demonstrate that Notch4 is highly expressed in the media of pulmonary vascular and is upregulated in lung tissues from patients with HPH and HPH rats compared with control groups. In vitro, hypoxia induces the high expression of *Delta-4* and Notch4 in HPASMCs. The increased expression of Notch4 promotes HPASMCs proliferation and migration and inhibits cells apoptosis via ERK, JNK, P38 signaling pathways. Furthermore, co-immunoprecipitation result elucidates the interaction between Notch4 and ERK/JNK/P38. In vivo, silencing Notch4 partly abolished the increase in RVSP and pulmonary vascular remodeling caused by hypoxia in HPH rats.

**Conclusions:**

These findings reveal an important role of the Notch4-ERK/JNK/P38 MAPK axis in hypoxic pulmonary remodeling and provide a potential therapeutic target for patients with HPH.

**Supplementary Information:**

The online version contains supplementary material available at 10.1186/s12931-022-01927-9.

## Background

Pulmonary hypertension (PH) is a pathophysiological disorder characterized by increased pulmonary vascular resistance and pulmonary arterial pressure [1]. Pulmonary vascular remodeling is an important contributor to increased pulmonary vascular resistance. The process of pulmonary vascular remodeling involves excessive proliferation, migration, and apoptosis resistance of pulmonary artery smooth muscle cells (PASMCs) in the media of the pulmonary artery [2, 3]. The patients with PH due to chronic lung disease and/or hypoxia are classified as group III PH, mainly secondary to chronic obstructive pulmonary disease, interstitial lung disease, and so on [4]. These patients complicated with PH are usually diagnosed at end-stage with a poor prognosis. However, the benefits of current treatment on the prognosis of those patients remain limited. So far, the pathogenesis of hypoxic pulmonary vascular remodeling has not been fully elucidated.

The Notch4 protein is a member of the Notch family, a set of four transmembrane proteins (Notch1-4) that share a similar structure, but differ in their cellular localization and tissue distribution [5]. In all members of the Notch receptors, ligand binding triggers proteolytic receptor cleavage to release the Notch intracellular domain. Then the Notch intracellular domain translocates to the nucleus where it binds to the CSL family of DNA-binding proteins and forms a transcriptional activator to activate Notch target genes [6–10]. Notch4 serves as a membrane-bound receptor that regulates cell fate [11]. Notch4 activation in endothelial cells promotes epithelial-mesenchymal transition, which is required for normal endocardial cushion differentiation and vascular smooth muscle cell development [12]. In addition, Notch4 plays an important role in promoting carcinogenesis and metastasis in several types of tumors [13–15]. However, the role of Notch4 in the smooth muscle cells remains unclear.

In certain context, Notch crosstalk with numerous pathways, such as Akt, TGF-β and src signaling [16–18]. Protein phosphorylation is one of the most important post-translational modifications regulating various signaling transduction. Mitogen-activated protein kinase (MAPK) members including ERK, JNK, and P38 kinases [19, 20]. These serine-threonine protein kinases play critical roles in the regulation of many cellular processes including cell cycle, proliferation, and apoptosis [21, 22]. Previous studies have shown that hypoxia promoted cell proliferation and inhibited apoptosis of PASMCs and sequentially induced pulmonary vascular remodeling via phosphorylated activation of ERK, JNK, and P38 signaling pathways [23–25]. Several studies have demonstrated that Notch4 was involved in the regulation of survival and migration of tumor and endothelial cells via MAPK signaling pathways [26–29]. This study aims to elucidate the role and molecular mechanism of Notch4 in hypoxic pulmonary arterial smooth muscle cells through in vitro and in vivo models.

## Methods

### Clinical samples

A group of surgical lung tissues were collected from 4 PH patients including 2 patients with PH secondary to chronic obstructive pulmonary disease and 2 patients with PH related to interstitial lung disease, and 4 patients without PH in the control group. According to the previous method in the study on PH [30], lung tissues from the control patients were commonly collected at a site remote from tumor foci.

All patients were enrolled from Tongji Hospital of Huazhong University of Science and Technology (Wuhan, China). This study was approved by the Ethics Committee of Tongji Hospital. Written informed consent was obtained from each patient.

### Cell lines and cultures

HPASMCs were obtained from Procell Life Science & Technology Corporation. All cells were cultured in Dulbecco's Modified Eagle Medium supplemented with 10% fetal bovine serum and maintained in a humidified incubator with 5% CO_2_ at 37 °C. The hypoxic HPASMCs were cultured in a 5% O_2_ incubator (Galaxy R; RS Bitotech, Alloa, UK) continually gassed with 5% CO_2_ and 90% N_2_.

### Antibodies and chemicals

The antibodies against proliferating cell nuclear antigen (PCNA), Bcl-2 associated X (Bax), survivin, matrix metallopeptidase 9 (MMP9), matrix metallopeptidase 2 (MMP2), β-Actin and normal rabbit or mouse immunoglobulin G (IgG) were obtained from Proteintech (Wuhan, Hubei, China). B cell leukemia/lymphoma 2 (Bcl-2) antibody was obtained from Boster (Wuhan, Hubei, China). P38, p-P38, JNK, p-JNK, ERK, p-ERK antibodies, and Protein G magnetic beads were obtained from Cell Signaling Technology (Danvers, MA, USA). Anti-Notch4 was obtained from Santa Cruz Biotechnology (Dallas, Texas, USA). HRP-conjugated anti-Rabbit IgG and HRP-conjugated anti-Mouse IgG were obtained from Servicebio (Wuhan, Hubei, China). HRP-conjugated anti-Rabbit IgG light chain and HRP-conjugated anti-mouse IgG light chain were obtained from Abbkine Scientific (Redlands, CA, USA). U0126, SP600125 and SB203580 were obtained from MedChemExpress (Monmouth Junction, NJ, USA).

### Cell transfection

Small interfering RNAs (siRNAs) against Notch4 and negative control siRNA were synthesized by RiboBio (Guangzhou, China). HPASMCs were transfected with siRNAs using Lipofectamine 3000 (Invitrogen, Carlsbad, CA) according to the manufacture’s instruction. The siRNA against Notch4 sequence was as follows: CAACGGGCACUGUGAGAAA. When reaching 40–60% of confluence, the cells were transfected with 50 nmol siRNA using Lipofectamine 3000. Notch4 overexpression plasmid and negative control plasmid were synthesized by GeneChem (Shanghai, China). The mammalian expression plasmid for Flag-tagged Notch4 was constructed by molecular cloning. The construct was confirmed by DNA sequencing. When reaching 80–90% of confluence, the cells were transfected with 1 μg purified plasmid. Finally, the mRNA and protein levels of cells were analyzed by quantitative real-time PCR and western blotting at 48 h and 72 h after transfection, respectively.

### Cell viability assay

Cell viability was measured using the Cell Counting Kit-8 assay. HPASMCs were seeded into 96-well plates at the cell density of 3000 cells per well for 24 h. Then the medium was replaced with different transfection mixture. After 6 h incubation, the transfection mixture was replaced with medium supplemented with 10% fetal bovine serum, and the cells were further incubated for 24 h. After that, cells were incubated under normoxic or hypoxic conditions for 24 h. Finally, 10 μL Cell Counting Kit-8 (Dojindo, Japan) reagent was added into per well and optical density in each well was determined by an ELx800 Universal Microplate Reader (BioTek Instruments, Winooski, VT, USA).

### Edu proliferation assay

HPASMCs were seeded into 96-well plates (3000 cells per well) and cultured in normoxic or hypoxic conditions as mentioned above. Cell proliferation was assessed using Edu Cell Proliferation Assay Kit (RiboBio, Cell‐Light™ Edu Apollo^®^643 In Vitro imaging kit), according to the manufacture’s instruction. The cells were added with 100 μL Edu (diluent reagent A with a complete medium by 1:1000) and incubated for 2 h at 37 °C. Then cells were fixed in 4% paraformaldehyde for 15–30 min and incubated with 1 × Apollo^®^ solution for another 30 min at room temperature. Cell nuclei were stained with 100 μL 1 × Hoechst33342 for 30 min. Finally, the cells were examined under a fluorescent microscope (Olympus, Japan). Data were shown as fold-change increase in the percentage of Edu-incorporating cells in treated cells, compared with negative controls.

### Cell apoptosis assay

HPASMCs were seeded into a 6-well plate (100,000 cells per well). Firstly, cells were starved in serum-free for 24 h. After transfection for 48 h, cells were exposed to normoxic or hypoxic conditions for another 24 h. Then cells were collected and resuspended in 200 μL binding buffer per well. The cells were labeled with 5 μL Annexin-V and propidium iodide (PI) to assess cell apoptosis using an Annexin-V/PI detection kit (Keygen Biotech, Nanjing, China). Finally, apoptotic cells were analyzed using flow cytometry (BD Biosciences, SanJose, CA). Apoptotic cells including early apoptotic cells (Annexin-V positive and PI-negative) and late apoptotic cells (Annexin-V positive and PI-positive) were shown.

### Cell migration assay

Cell migration assay was performed in a 24-well plate (Corning, MA, USA) according to the manufacture’s instruction. Firstly, 200 μL cell suspensions (10,000 cells totally) containing 1% fetal bovine serum were seeded into the upper chamber, while 600 μL medium containing 15% fetal bovine serum were added into lower chambers. The cells were cultured in normoxic or hypoxic condition for 24 h. Then cells on the lower surface of the membrane were fixed with 4% paraformaldehyde and stained with 0.1% crystal violet. Non-migrated cells were removed by scraping the membranes with a cotton swab from the upper surface. Finally, cells were counted under an optical microscope (Zeiss, Oberkochen, Germany).

### RNA extraction and real-time quantitative PCR

Total RNA was isolated using Trizol (Takara, Dalian, China) according to the manufacture’s instruction. RNA concentration was determined by Nanodrop analysis. cDNA was synthesized from total RNA using PrimeScript RT reagent kit (Takara, Dalian, China). Quantitative RT-PCR was performed using SYBR Green Mix (Takara, Dalian, China). The primer sequences were as follows: Notch4 forward, 5′-CGTACCCCACTTCACACTGC-3′, reverse, 5′-AGGTGTAGTCCCGTCGTCTG-3′.

The cycling conditions were as follows: initial denaturation for 10 min at 95 °C followed by 40 cycles of denaturation (15 s at 95 °C), annealing and elongation (30 s at 60 °C). The relative expression of gene was calculated using 2^−△△Ct^ method using β-Actin as the reference gene.

### Western blotting analysis

Total cellular protein was extracted by RIPA lysis buffer (50 mM Tris, 150 mM NaCl, 1% NP-40, 0.5% sodium deoxycholate, 0.1% SDS, PH7.4) supplemented with phenylmethylsulfonyl fluoride, cocktail, and phosphorylation protease inhibitor. Cell lysis was centrifuged at 12,000 rpm for 15 min and the supernatants were collected for determination of the protein concentration by BCA assay. All steps were performed at 4 °C. Then 50 μg of protein were subjected to 10% sodium dodecyl sulfate–polyacrylamide gel electrophoresis followed by western blotting. The signals were detected using a chemiluminescent substrate system (Bio-Rad, Hercules, CA, USA). Relative expressions of target proteins were quantified by Image J software.

### Co-immunoprecipitation assay

Co-immunoprecipitation experiments were performed according to a standard protocol. Briefly, cells were harvested and lysed in IP-lysis buffer (50 mM Tris, 150 mM NaCl, 1% NP-40) and protease inhibitor. Supernatants were collected by microcentrifuge (14,000 rpm, 10 min, 4 °C), and pre-cleared with 20 μL Protein G Magnetic beads (Cell Signaling Technology) for 2 h. Then the pre-cleared supernatants were incubated with primary antibodies (2 μg/mL) rotated overnight at 4 °C to form an immunocomplex. Then transferred immunocomplex solution to the tube containing pre-washed magnetic beads and incubated rotated for 3–4 h at 4 °C. Finally, the immunocomplex was washed five times with 500 μL of 1 × cell lysis buffer and analyzed by western blotting analysis.

### Immunohistochemistry

Formalin-fixed, paraffin-embedded tissue sections were de-waxed and antigen retrieval with citrate buffer (PH = 6.0). Then sections were incubated in 3% hydrogen peroxide for 20 min at room temperature to block endogenous peroxidase, followed by blocked in 5% bovine serum for 40 min. Primary antibodies were added to sections and incubated overnight at 4 °C in a humidified chamber, then incubated with secondary antibody for 40 min at room temperature. For immunohistochemistry analysis, the sections were further incubated with the avidin–biotin-peroxidase complex, which was detected with diaminobenzidine. Finally, sections were counterstained by hematoxylin, dehydrated in graded ethanol, and mounted with Permount.

### Immunofluorescence

Frozen lung sections were fixed with 4% paraformaldehyde for 15 min. Primary antibodies were added to sections and incubated overnight at 4 °C in a humidified chamber after blocked with 5% donkey serum albumin for 1 h. Alexa 594-labeled anti-mouse and Alexa 488-conjugated anti-rabbit antibody (Invitrogen, CA, USA) were used as secondary antibodies. Nuclei were counterstained with DAPI for 5 min.

### Animal experiments

All animal experiments were performed in compliance with the guidelines for animal testing and research, with ethical approval from Tongji Hospital. Adult male Sprague–Dawley rats weighing 200–250 g were fed standard chow and water ad libitum. The rats were exposed to either normoxia or chronic hypoxia (10% O_2_) 8 h daily for 5 weeks with a 12 h light–dark cycle. Hypoxic rats were randomly divided into four groups: hypoxic group (n = 5); AAV1-si-NC group (100 μL, n = 5); AAV1-si-Notch4 prophylactic group (100 μL, n = 7); AAV1-si-Notch4 therapeutic group (100 μL, n = 6). Hypoxic therapeutic group were intratracheal delivery of AAV1-si-Notch4 after exposed to a hypoxic atmosphere for two weeks. In the prophylactic group, AAV1-si-Notch4 was delivered at an equivalent dose to rats before exposed to the hypoxic chamber. At 5 weeks, rats were anesthetized with 1% pentobarbital injection (120 mg/kg ip). RVSP was measured via the right jugular vein using a 1.2-F pressure catheter. Then hearts and pulmonary vasculature were perfused with cold saline injection into the right ventricle. Hearts were excised and measured by Fulton’s Index (ratio of right ventricle weight to left ventricle plus septum weight, RV/[LV + S]). Right lungs were snap-frozen in liquid nitrogen and stored at – 80 °C for biochemical measurements. Left lungs were perfused with 4% paraformaldehyde, followed by paraffin embedding. Sections were stained with hematoxylin and eosin and immunohistochemistry for α-smooth muscle actin. The medial wall thickness index was calculated as follows: Wall thickness (%) = 100 × (external diameter − internal diameter)/external diameter.

### Statistics

Statistical analyses were performed using GraphPad Prism version 8.0. Results were plotted as means ± SD. The Student’s *t*-test was used for two-sample analyses, and normal distributions were assumed. One-way analysis of variance with Tukey’s post hoc test was used for more than two-sample analyses. All experiments were performed at least in triplicate. Significance was considered at the level of *P* < 0.05.

## Results

### Notch4 expression is upregulated in HPH patients and HPASMCs under hypoxia

**Fig. 1:**
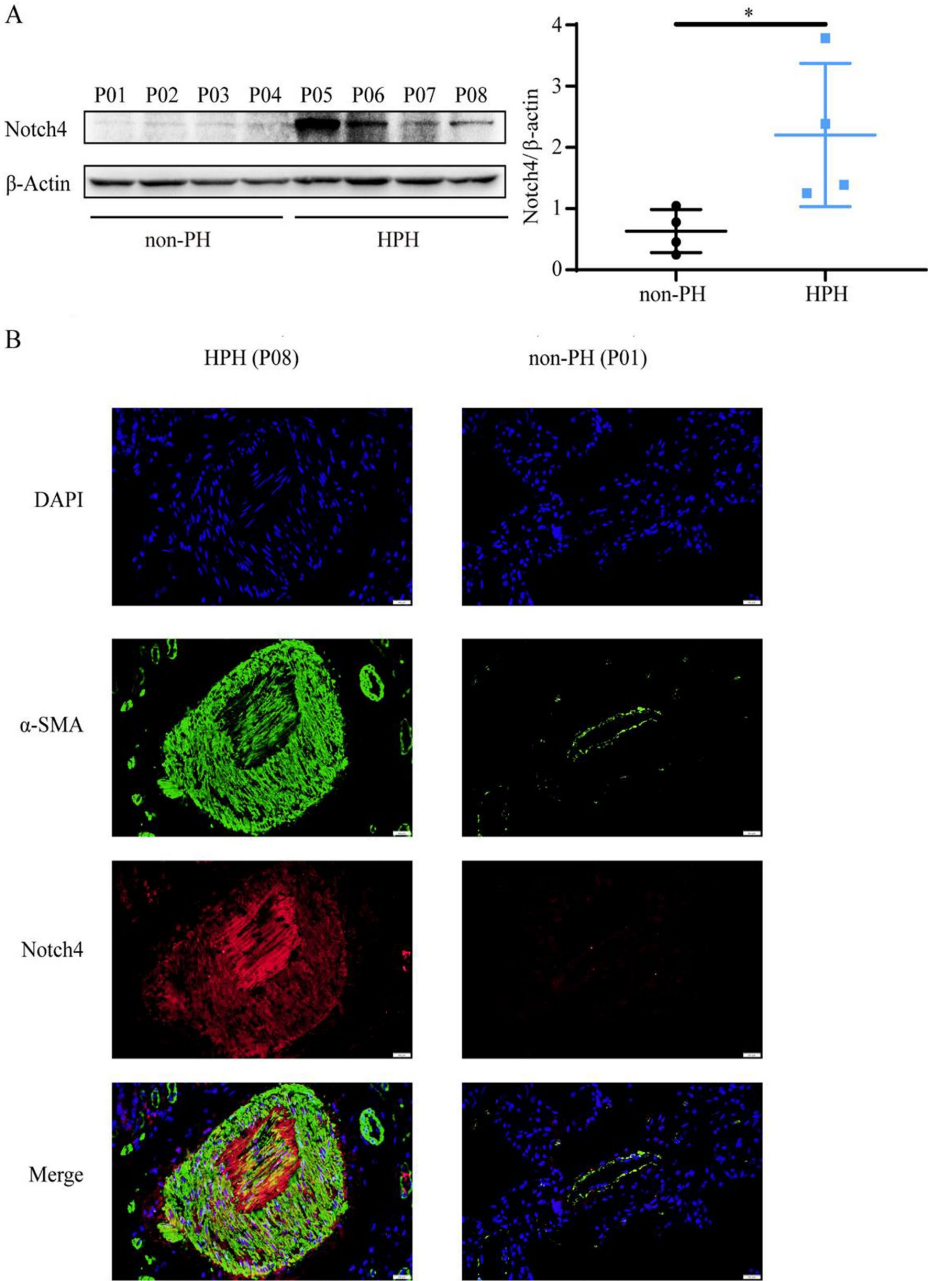
Notch4 expression is upregulated in lung tissues from patients with HPH and controls. Lung tissues were obtained from patients with confirmed HPH (n = 4) and matched control non-PH patients. (n = 4) **A** Protein levels of Notch4 in lung tissues from patients with HPH and without PH. **B** Notch4 (red) and α-SMA (green) immunofluorescence staining in small pulmonary arteries from humans with HPH (P08) or non-PH (P01). Nuclei are counterstained with DAPI (blue). Magnification, × 400; Bar, 20 μm. Data were presented as means ± SD. *P < 0.05. Student’s t-test was performed for **A**. PH, pulmonary hypertension; HPH, hypoxic pulmonary hypertension; α-SMA, α-smooth muscle actin

To investigate the expression of Notch4 in normal and pulmonary hypertensive lungs, we examined lung tissues from 4 individuals with HPH undergoing lung transplantation compared with lung tissues from 4 non-PH individuals (Tables 1, 2). The protein levels of Notch4 had a tendency to increase in HPH patients compared with non-PH patients (Fig. 1A). Immunofluorescent staining of human lung tissues showed that Notch4 was localized in the media and intimal layer of small pulmonary arterials. Higher level of Notch4 staining was seen in lung tissues with HPH compared to matched control lung tissues (Fig. 1B). Vascular smooth muscle cells (vSMCs) are the predominant constituents of media. The excessive vSMCs proliferation, migration and apoptosis resistance plays a key role in vascular remodeling in HPH. Therefore, we focus on the function and the underlying mechanisms of Notch4 in HPASMCs isolated from human small pulmonary arteries. Analysis of cultured HPASMCs exposed to normoxia or hypoxia demonstrated that Notch4 mRNA and protein levels were increased under hypoxic conditions and peaked at 3 h and 24 h, respectively (Additional file 1: Fig. S1A and S1B). Meanwhile, increased mRNA level of *Delta-like 4* was also observed in HPASMCs exposed to hypoxia (Additional file 1: Fig. S1D), which may hint that hypoxia induces Notch4 signaling by increasing the levels of *Delta-like 4* in HPASMCs.

**Table 1:** Clinical characteristic of control patients

	Sex	Diagnosis
Patient 1 (P01)	Female	Adenocarcinoma
Patient 2 (P02)	Female	Adenocarcinoma
Patient 3 (P03)	Male	Adenocarcinoma
Patient 4 (P04)	Female	Adenocarcinoma

**Table 2:** Clinical characteristic of HPH patients

	Sex	Diagnosis	Date of LTx	Age at LTx	Pre-LTx mPAP (mmHg)	PG (mmHg)
Patient 5 (P05)	Female	HPH	2015/07/29	28	NA	NA
Patient 6 (P06)	Male	HPH	2015/07/04	59	NA	34
Patient 7 (P07)	Male	HPH	2019/06/02	65	NA	35
Patient 8 (P08)	Male	HPH	2021/08/17	57	NA	NA

*LTx* lung transplant, *mPAP* mean pulmonary arterial pressure, *PG* tricuspid regurgitation pressure gradient, *HPH* hypoxic pulmonary arterial hypertension

### Notch4 signaling is required for hypoxia-induced HPASMCs excessive proliferation, migration and apoptosis resistance

**Fig. 2:**
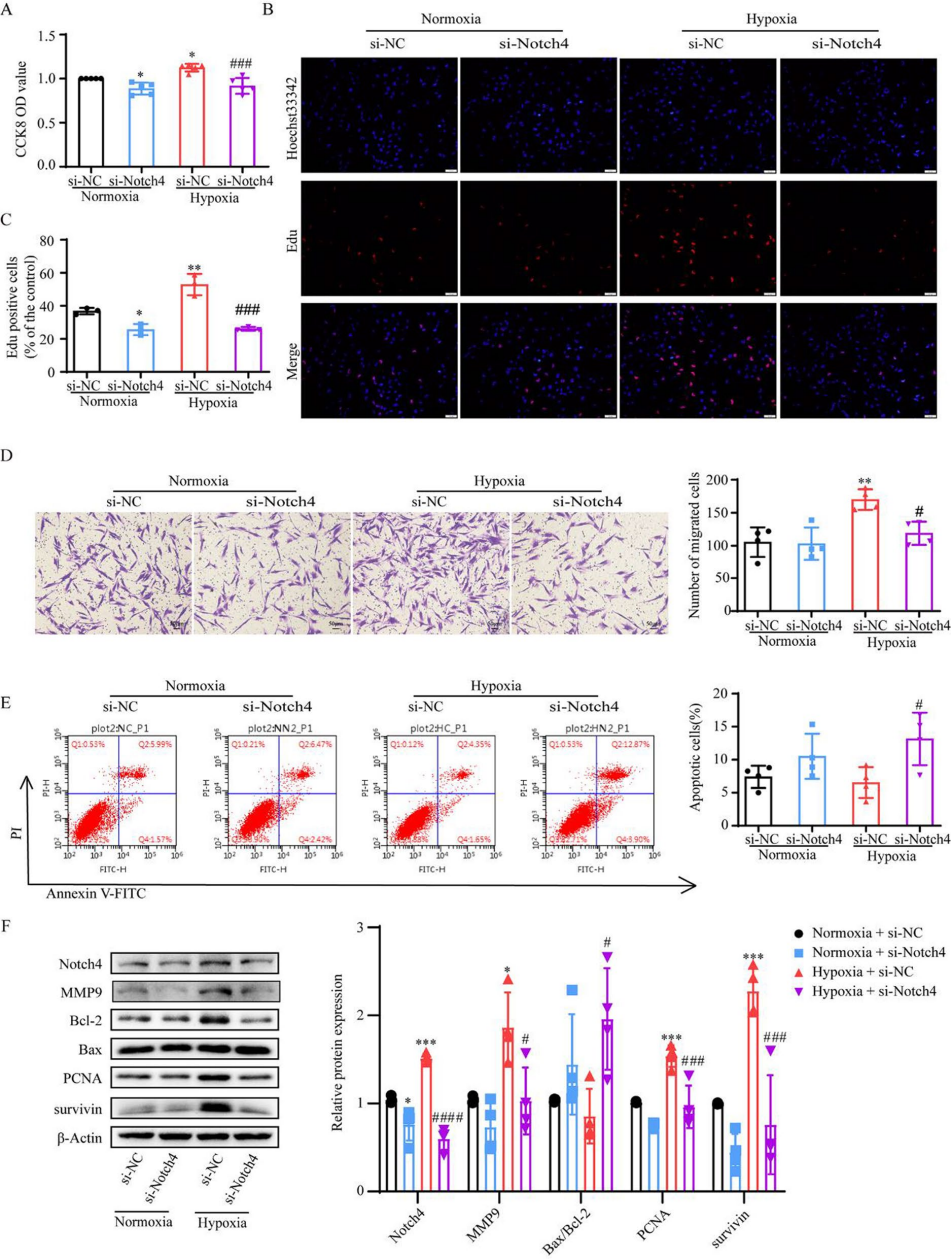
Notch4 signaling contributes to hypoxia-induced HPASMCs proliferation, migration and apoptosis resistance.** A** Cell viability was performed using Cell Counting Kit-8 assay in HPASMCs. (n = 5) **B, C** Cell proliferation was assessed by Edu assay. (n = 3) Magnification, × 100; Bar, 100 μm. **D** Cell migration was performed using Transwell assay. Magnification, × 100; Bar, 50 μm. (n = 4) **E** Cell apoptosis was assessed by Annexin-V/propidium iodide (PI) staining. Analyses of apoptosis including early apoptosis (Annexin-V positive and PI negative) and late apoptosis (Annexin-V positive and PI positive) were shown. (n = 4) **F** Protein levels of Notch4, MMP9, Bcl-2, Bax, PCNA, survivin were shown. (n = 4) Data were presented as means ± SD. *P < 0.05, **P < 0.01, ***P < 0.001, comparison with normoxic HPASMCs treated with si-NC; ^#^*P* < 0.05, ^###^*P* < 0.001, ^####^*P* < 0.0001, comparison with hypoxic HPASMCs treated with si-NC. One-way ANOVA followed by Tukey’s correction for post-hoc comparisons were performed for **A–F**. *CCK-8* cell counting kit-8, *si-NC* negative control short interfering RNAs (siRNA), *si-Notch4* siRNA against Notch4

In our study, hypoxia promoted HPASMCs proliferation and migration, and inhibited cell apoptosis. To assess the role of Notch4 involved in the process in HPASMCs, siRNA against Notch4 were transfected into HPASMCs. The mRNA and protein expression of Notch4 were dramatically decreased following transfection with siRNA against Notch4 in HPAMSCs (Additional file 1: Fig. S2). The knockdown of Notch4 partially abrogated the excessive cell proliferation and migration, and suppressed cell apoptosis resistance under hypoxia (Fig. 2A–E). The levels of proteins associated with cell proliferation (survivin and PCNA), apoptosis (Bax and Bcl-2), and migration (MMP9) were measured in HPASMCs transfected with si-Notch4 or si-NC. Consistently, up-regulation of survivin, PCNA, MMP9, and decreased ratio of Bax/Bcl-2 were observed in hypoxic HPASMCs and partially abrogated by transfection with siRNA against Notch4 (Fig. 2F). Taken together, these findings indicate that hypoxia-induced HPASMCs proliferation, migration and apoptosis resistance involves Notch4 signaling.

### ERK, JNK, and P38 signaling pathways mediate the regulation of Notch4 on HPASMCs proliferation, apoptosis, and migration

**Fig. 3:**
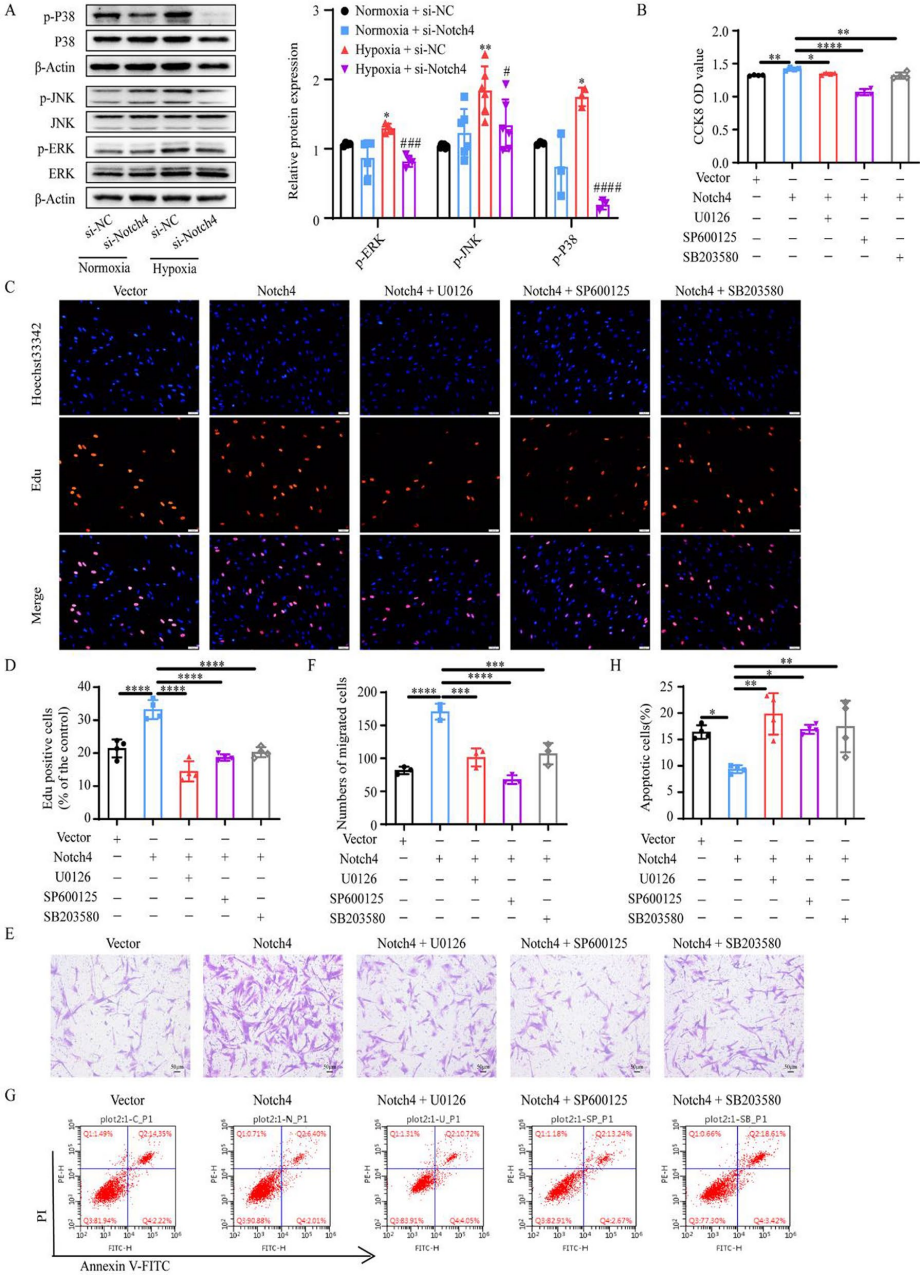
ERK, JNK, and P38 MAPK signaling mediate the regulation of Notch4 on HPASMCs proliferation, apoptosis, and migration. **A** The MAPK signaling pathway were detected in HPASMCs transfected with siRNA against Notch4 or negative control siRNA. (n = 3–6) **B** Cell viability was examined in HPASMCs transfected with Notch4 plasmid or negative control plasmid together with ERK (U0126), JNK (SP600125), or P38 (SB203580) MAPK pathway inhibitors by using Cell Counting Kit-8 assay. (n = 4) **C, D** Proliferation was assessed by Edu assay in HPASMCs transfected with Notch4 plasmid or negative control plasmid together with U0126 (15 μM), SP600125 (15 μM), or SB203580 (15 μM) for 24 h. (n = 4) Magnification, × 100; Bar, 100 μm. **E, F** Migration was determined in HPASMCs transfected with Notch4 plasmid or negative control plasmid together with U0126, SP600125, or SB203580 for 24 h. Magnification, × 100; Bar, 50 μm. (n = 3) **G, H** The Annexin-V/PI assay was performed in HPASMCs transfected with Notch4 plasmid or negative control plasmid together with U0126, SP600125, or SB203580 for 24 h. Analyses of apoptosis including early apoptosis (Annexin-V positive and PI negative) and late apoptosis (Annexin-V positive and PI positive) were shown. (n = 4) Data were presented as means ± SD. *P < 0.05, **P < 0.01, ***P < 0.001, ****P < 0.0001, comparison with normoxic HPASMCs treated with si-NC; ^#^*P* < 0.05, ^##^*P* < 0.01, ^###^*P* < 0.001, comparison with hypoxia HPASMCs treated with si-NC. One-way ANOVA followed by Tukey’s correction for post-hoc comparisons were performed for **A**–**H**. *CCK-8* cell counting kit-8, *ERK* extracellular signal-regulated kinase, *JNK* c-Jun N-terminal kinase, *MAPK* mitogen-activated protein kinase, *si-NC* negative control short interfering RNAs (siRNA), *si-Notch4* siRNA against Notch4, *vector* negative control plasmid, *Notch4* Notch4 plasmid

**Fig. 4:**
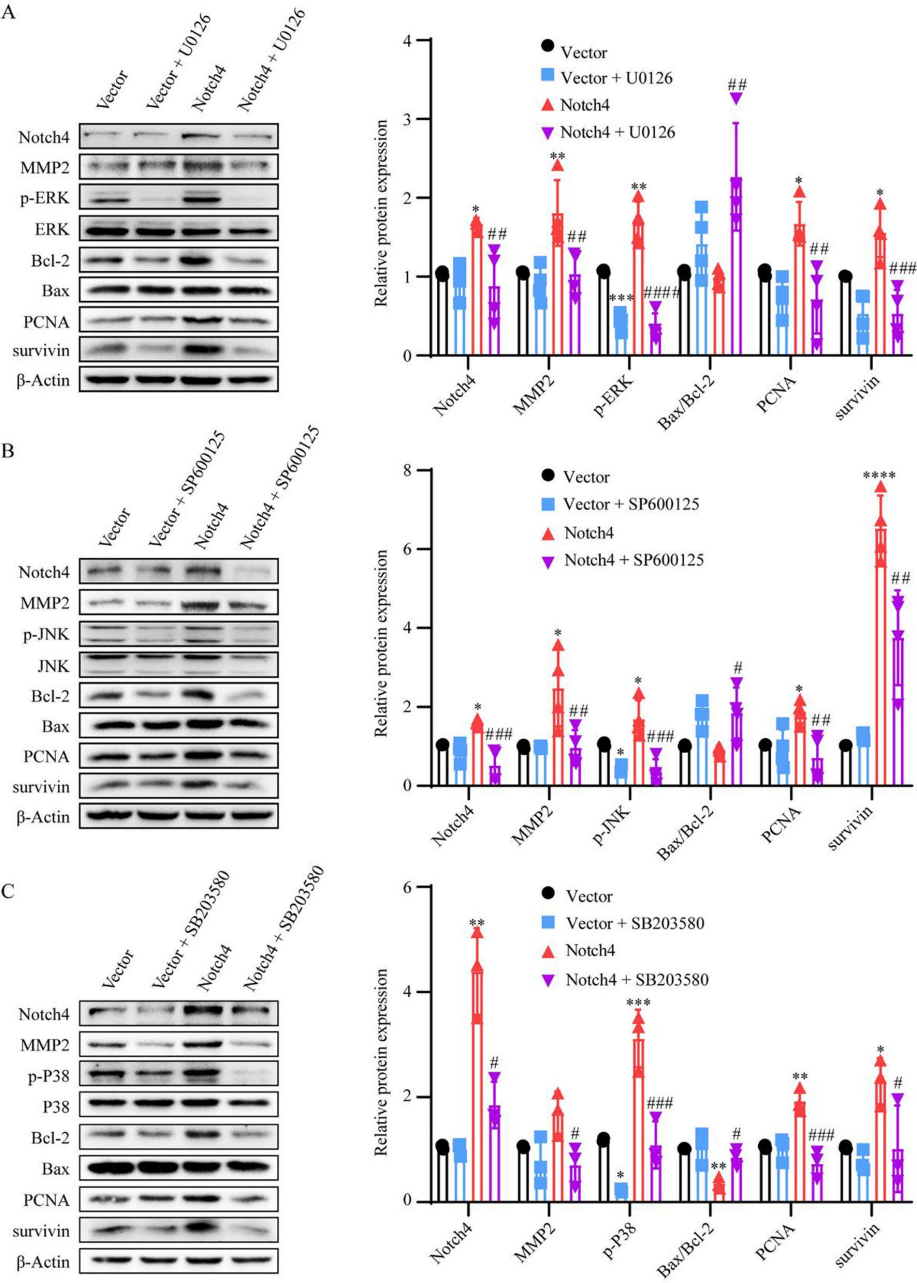
The regulation of Notch4 on the protein levels associated with proliferation, apoptosis and migration through ERK, JNK, or P38 MAPK pathway. **A** Protein levels of survivin, PCNA, Bax, Bcl-2, ERK, p-ERK, MMP2, and Notch4 were determined in HPASMCs transfected with negative control plasmid or Notch4 plasmid together with U0126 for 24 h. Bars show mean fold change of vector or as indicated. (n = 4) **B** Protein levels of survivin, PCNA, Bax, Bcl-2, JNK, p-JNK, MMP2, MMP9, and Notch4 were determined in HPASMCs transfected with negative control plasmid or Notch4 plasmid together with SP600125 for 24 h. Bars show mean fold change of vector or as indicated. (n = 4) **C** Protein levels of survivin, PCNA, Bax, Bcl-2, JNK, p-JNK, MMP2, and Notch4 were determined in HPASMCs transfected with negative control plasmid or Notch4 plasmid together with SB203580 for 24 h. Bars show mean fold change of vector or as indicated. (n = 3) Data were presented as means ± SD. *P < 0.05, **P < 0.01, ***P < 0.001, ****P < 0.0001, comparison with HPASMCs transfected with negative control plasmid; ^#^*P* < 0.05, ^##^*P* < 0.01, ^###^*P* < 0.001, ^####^*P* < 0.0001, comparison with HPASMCs transfected with Notch4 plasmid. One-way ANOVA followed by Tukey’s correction for post-hoc comparisons were performed for **A**–**C**. *ERK* extracellular signal-regulated kinase, *JNK* c-Jun N-terminal kinase, *MAPK* mitogen-activated protein kinase, *vector* negative control plasmid, *Notch4* Notch4 plasmid

**Fig. 5:**
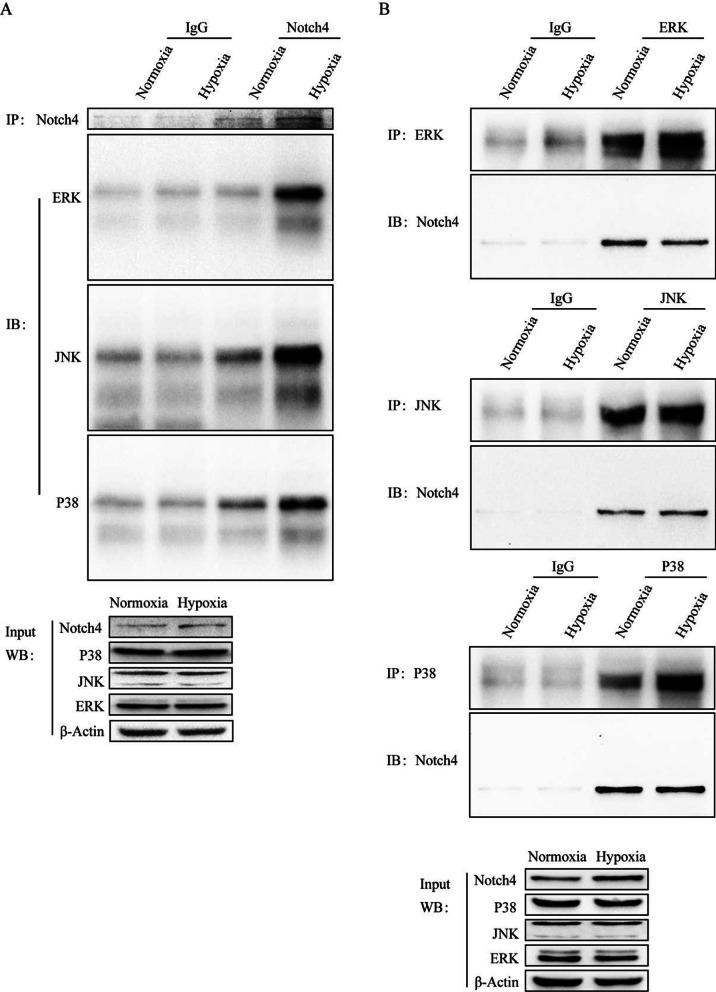
Notch4 interacts with ERK, JNK, and P38 in HPASMCs. Endogenous co-immunoprecipitation was performed in HPASMCs exposed to normoxia or hypoxia for 24 h. *ERK* extracellular signal-regulated kinase, *JNK* c-Jun N-terminal kinase

The experiments described above implicate that Notch4 signaling mediate the effect of hypoxia on HPASMCs proliferation, apoptosis, and migration. We next explored the molecular mechanisms in the process. The level of Notch4 overexpression was determined by qRT-PCR and Western blotting (Additional file 1: Fig. S3). Notch4 overexpression promoted HPASMCs proliferation and migration, and inhibited cell apoptosis (Fig. 3B–H). The interaction between Notch4 and MAPK pathway in hypoxia HPASMCs was investigated. Hypoxia upregulated the phosphorylation of ERK, JNK, and P38 in HPASMCs, which were partially abrogated by transfection of siRNA against Notch4 (Fig. 3A). Meanwhile, the specific inhibitors of ERK, JNK, and P38 pathways (U0126, SP600125, or SB203580) partially abrogated the excessive proliferation and migration, and apoptosis resistance of HPASMCs induced by Notch4 overexpression (Fig. 3B–H). Furthermore, we assessed the expression of proteins associated with cell proliferation, apoptosis, and migration. Consistently, Notch4 overexpression increased protein expressions of survivin, PCNA, MMP2, and decreased the ratio of Bax/Bcl-2, which were partly restored by co-treatment with U0126, SP600125, or SB203580 (Fig. 4A–C). Taken together, our results demonstrate that MAPK pathway is responsible for mediating the effect of Notch4 on HPASMCs proliferation, apoptosis, and migration under hypoxia. The interaction between Notch4 and ERK/JNK/P38 was confirmed using co-immunoprecipitation. The above results provide some clues for a novel crosstalk mechanism between non-canonical Notch signaling and MAPK pathway via interaction between Notch4 and ERK/JNK/P38 (Fig. 5A and B).

### Notch4 gene silence improves the hemodynamic parameters in HPH rats

**Fig. 6:**
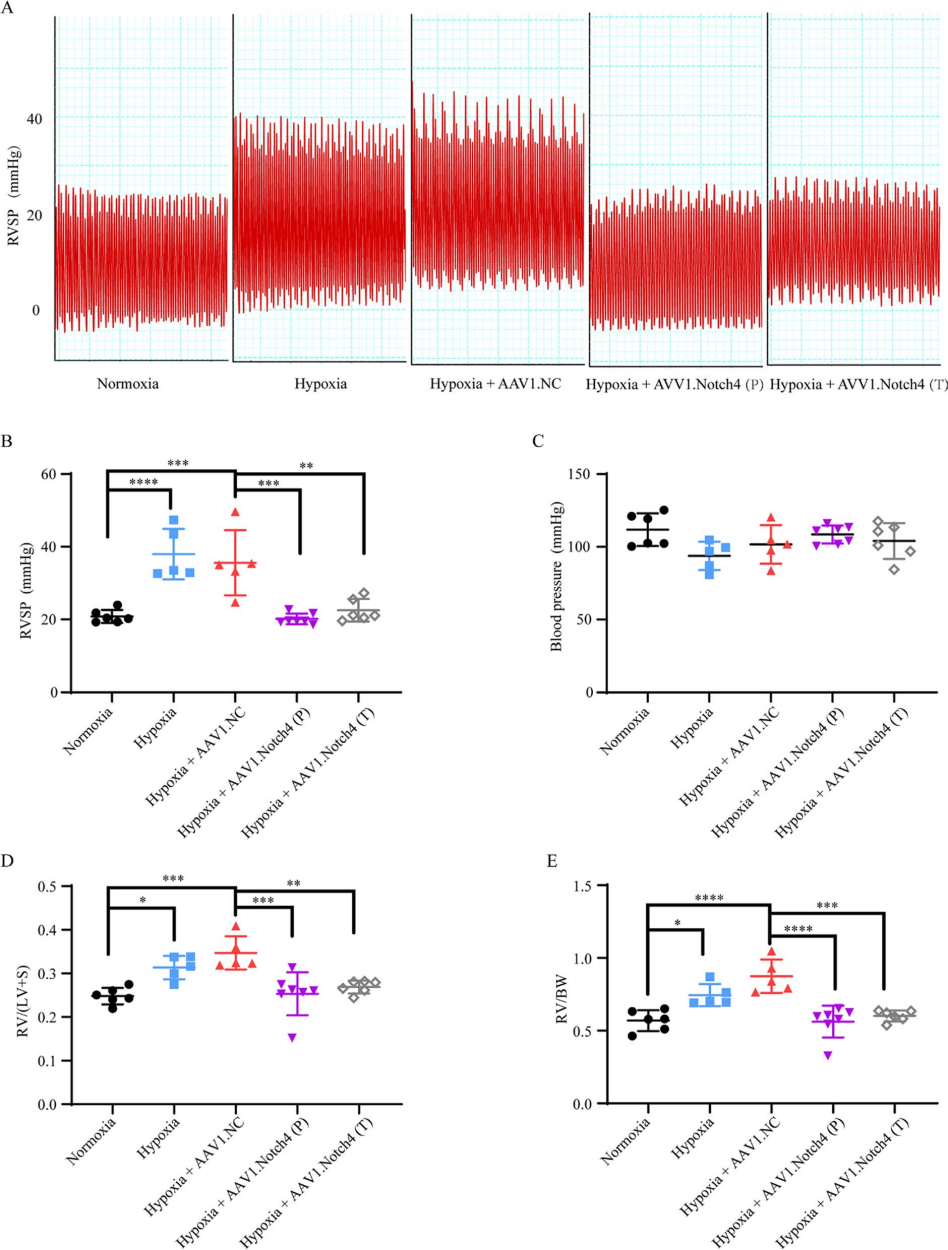
Intratracheal delivery of AAV1.Notch4 improves pulmonary hemodynamics in the hypoxic PH rats compared with negative controls. **A, B** The preventive and therapeutic efficacy of AAV1.Notch4 were assessed by right ventricular systolic pressure (RVSP). **C** Blood pressure (BP) were measured in rats treated with AAV1.Notch4 or AAV1.NC under normoxic or hypoxic condition. **D** RV hypertrophy was determined by the RV/(LV + S) weight ratio. **E** RV hypertrophy was determined by the RV/BW ratio. Data were presented as means ± SD. *P < 0.05, **P < 0.01, ***P < 0.001, ****P < 0.0001. One-way ANOVA followed by Tukey’s correction for post-hoc comparisons were performed for **B**–**E**. *AAV* adeno-associated virus, *PH* pulmonary hypertension, *RVSP* right ventricular systolic pressure, *RV* right ventricle, *LV* left ventricle, *BW* body weight, *S* septum, *P* prevention, *T* treatment

**Fig. 7:**
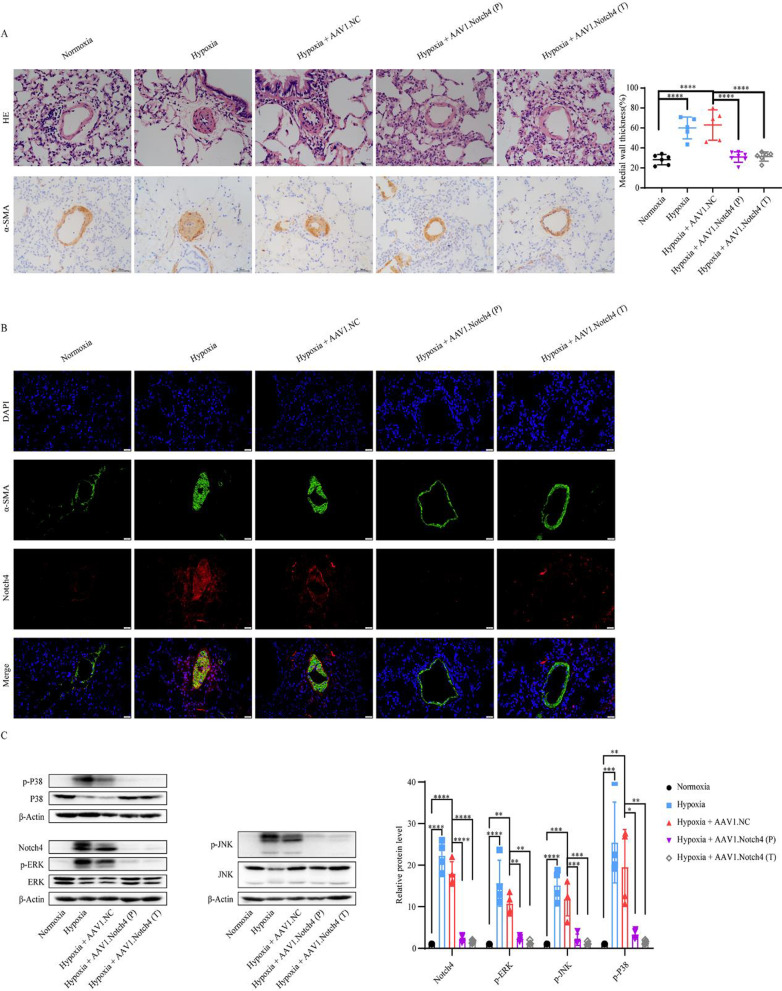
Notch4 gene silence alleviates pulmonary artery remodeling. **A** Hematoxylin–eosin staining and immunohistochemistry staining for α-smooth muscle actin, in lung sections of rats treated with AAV1.Notch4 or AAV1.NC under normoxic or hypoxic condition (n = 5–7). Magnification, × 200; Bar, 50 μm. **B** Notch4 (red) and α-SMA (green) immunofluorescence staining in small pulmonary arteries in rat lung tissues. Nuclei are counterstained with DAPI (blue). Magnification, × 400; Bar, 20 μm. **C** Protein expression of Notch4, p-ERK, ERK, p-JNK, JNK, p-P38, P38 in pulmonary artery in rats treated with AAV1.Notch4 or AAV1.NC under normoxic or hypoxic condition (n = 4). Data were presented as means ± SD. *P < 0.05, **P < 0.01, ***P < 0.001, ****P < 0.0001. One-way ANOVA followed by Tukey’s correction for post-hoc comparisons were performed for **A** and **C**. *H&E* hematoxylin–eosin staining, *α-SMA* α-smooth muscle actin, *AAV* adeno-associated virus, *ERK* extracellular signal-regulated kinase, *JNK* c-Jun N-terminal kinase

Our previous study has reported a rat model of pulmonary vascular remodeling exposed to hypoxia for 2 weeks [31]. An adeno-associated virus (AAV) 1- encoded si-Notch4 was intratracheally delivered into the rats on day 0 and week 2 after hypoxic exposure in the prophylactic group and therapeutic group, respectively. We showed that AAV1-si-Notch4 can cross biological barriers and effectively achieve targeted pulmonary vascular media (Additional file 1: Fig. S4) and vSMCs-specifically silence Notch4 (Fig. 7B). The results showed that silencing Notch4 significantly reduced the RVSP of HPH rats measured by right cardiac catheterization (Fig. 6A and B). There was no significant difference in systematic arterial blood pressure among all the groups (Fig. 6C).

### Notch4 gene silence prevents and attenuates pulmonary vascular remodeling in HPH rats

The prevention and attenuation of hypoxia-induced vascular remodeling by airway luminal delivery of AAV1-si-Notch4 was assessed by hematoxylin and eosin staining, immunohistochemistry staining for α-smooth muscle actin (Fig. 7A), RV hypertrophy assessed by RV/(LV + S) weight and RV/BW weight (Fig. 6D and E). Consistently, the protein levels of p-ERK, p-JNK, and p-P38 were decreased in the pulmonary artery in HPH rats treated with AAV1-si-Notch4 compared with negative controls (Fig. 7C).

## Discussion

Notch4 is located and upregulated in pulmonary vascular smooth muscle from patients with HPH and HPH rats. In vitro, hypoxia induces the high expression of *Delta-4* and Notch4 in HPASMCs. The increased expression of Notch4 promotes HPASMCs proliferation and migration and inhibits cells apoptosis via ERK, JNK, P38 signaling pathways. In vivo, AAV1-mediated gene silencing of Notch4 significantly reduced RVSP, and attenuated pulmonary vascular remodeling and RV hypertrophy in HPH rats. Our results establish a link between Notch4 signaling and MAPK pathway involved in PASMCs proliferation, apoptosis, and migration, and reveal a novel molecular regulatory axis that plays an essential role in the pathogenesis of HPH.

Notch receptors are critical for controlling vSMCs differentiation and phenotypic transformation [32], which contributes to vascular remodeling [33]. A study reported that the constitutive active intracellular domain of Notch1, Notch2, or Notch4 receptors induced smooth muscle alpha-actin expression [34]. In this study, Notch4 was localized in the media and intimal layer of pulmonary small artery and the expression of Notch4 in the media was upregulated in patients with HPH and HPH rats compared with controls, which supports for the role of Notch4 in the pathogenesis of hypoxic pulmonary vascular remodeling.

Hypoxia activates the notch signaling pathway in cancer cells and nonmalignant cell types (such as intervertebral disc cells, Müller cells and astrocytes) [35, 36]. Hypoxia inducible factor1-α, a mediator of oxygen sensing, has been found to interact with Notch intracellular domain and act as a co-enhancer to stimulate transcription of Notch target genes under hypoxic conditions [37]. In this study, we reported a high expression of Notch4 in PASMCs both in vivo and in vitro, consistent with the previous study [38]. Knockdown of Notch4 partially abrogated the excessive HPASMCs proliferation, migration, and apoptosis resistance due to hypoxia. Thus, Notch4 is required in the process of hypoxia-induced vascular remodeling.

Notch receptors can affect cell proliferation, apoptosis, and migration via interacting with other nuclear transcription factors (NF-κB [39], Smad3 [40]) or other intracellular signaling (Wnt/β-catenin [41], Akt [29]). MAPK signaling pathway, as an important intracellular signal transduction pathway, plays an important role in hypoxic pulmonary vascular remodeling. Activation of Notch1 signaling has been found to enhance MAPK activity via Notch1/MAML cascade-mediated transcription, and then induced melanoma cell growth [42]. In our study, in vivo and in vitro studies indicate that hypoxia upregulates Notch4 and activates ERK, JNK, and P38 pathways in hypoxic HPASMCs and rats pulmonary vascular. Knockdown of Notch4 suppressed the phosphorylation of ERK, JNK, and P38 in HPASMCs, and inhibited excessive cell proliferation and migration and induced cell apoptosis. Meanwhile, Notch4 knockdown partially reversed hypoxic pulmonary vascular remodeling. Furthermore, the MAPK inhibitors targeting ERK [43], JNK [44], and P38 [45] can significantly reverse the excessive proliferation and migration and inhibited apoptosis induced by Notch4 overexpression. The above results suggest that the MAPK signaling pathway mediates the regulation of Notch4 on HPASMCs proliferation, apoptosis, and migration. Moreover, Notch4 was observed to interact with ERK/JNK/P38 by co-immunoprecipitation assay.

The safety represents a significant concern in gene therapy. In recent years, recombinant AAV has attracted great interest as a gene therapy vector because of stable expression of transgene without disturbing the function of the host genome [46]. AAV1 has a relative tropism for the heart (cardiomyocytes), and the endothelium, and vascular smooth muscle [30]. In this study, we used an AAV1 vector via intratracheal injection based on our previous work which demonstrated efficient transduction of AAV1 in pulmonary vessels [47]. Here, the intratracheal delivery of AAV1-si-Notch4 has both preventive and therapeutic efficacy in HPH rats. The AAV1 was highly expressed in the pulmonary vascular media of rat lung tissues. Notch4 expression was also vSMC-specifically silenced in the AAV1-si-Notch4 group.

There are some limitations to this study. Firstly, the western blotting result revealed an increased tendency of Notch4 in the lung tissues from 4 patients with HPH compared with matched controls. The power of the data is limited due to the insufficient sample size. Lung tissues from more HPH patients are needed for verification. Secondly, although the delivery of recombinant AAVs is promising, we must acknowledge the challenges here. The barrier concerns the immune response against the recombinant AAVs capsid, and poses substantial challenges to effective and safe gene delivery [48, 49]. Thirdly, female rats are not included in our animal model. As Mair et al. has demonstrated that endogenous estrogen plays a causative role in the development of hypoxic pulmonary hypertension in female animal models [50]. In addition, the male rat models are widely used in the study of hypoxic pulmonary hypertension [51]. We investigated the role of Notch4 in the development of hypoxic pulmonary hypertension in the male rat models.

In conclusion, this study demonstrates an important role of the Notch4-ERK/JNK/P38 MAPK axis in hypoxic pulmonary remodeling and provides a potential therapeutic target for HPH patients.

## Conclusions

This study reveals an important role of the Notch4-ERK/JNK/P38 MAPK axis in hypoxic pulmonary remodeling and provides a potential therapeutic target for patients with HPH.

## Supplementary Information

**Additional file 1. ** Notch4 mediates vascular remodeling via ERK/JNK/P38 MAPK signaling pathways in hypoxic pulmonary hypertension.

## Data Availability

The datasets used and/or analyzed during the current study are available from the corresponding author on reasonable request.

## Abbreviations

HPH: Hypoxic pulmonary hypertension
PH: Pulmonary hypertension
vSMCs: Vascular smooth muscle cells
PASMCs: Pulmonary artery smooth muscle cells
HPASMCs: Human pulmonary artery smooth muscle cells
ERK: Extracellular signal-regulated kinase
JNK: C-Jun N-terminal kinase
MAPK: Mitogen-activated protein kinase
RVSP: Right ventricular systolic pressure
PCNA: Polyclonal antibodies against proliferating cell nuclear antigen
Bcl-2: B cell leukemia/lymphoma 2
Bax: BCL2 associated X
MMP9: Matrix metallopeptidase 9
MMP2: Matrix metallopeptidase 2
IgG: Immunoglobulin G
siRNA: Small interfering RNA
AAV: Adeno-associated virus
