# Supplementary material for: Notch4 mediates vascular remodeling via ERK/JNK/P38 MAPK signaling pathways in hypoxic pulmonary hypertension
Source: Respir Res. 2022 Jan 11;23:6. doi: 10.1186/s12931-022-01927-9 (PMC8753901; doi:10.1186/s12931-022-01927-9)
Supplement: Supplementary file 1 — Additional file 1. Notch4 mediates vascular remodeling via ERK/JNK/P38 MAPK signaling pathways in hypoxic pulmonary hypertension. [file 12931_2022_1927_MOESM1_ESM.docx]

**SUPPLEMENTAL MATERIAL**

**Notch4 mediates vascular remodeling via ERK/JNK/P38 MAPK signaling pathways in hypoxic pulmonary hypertension**

Mingzhou Guo^1,2^, Mengzhe Zhang^1,2^, Xiaopei Cao^3^, Xiaoyu Fang^1,2^, Ke Li^1,2^, Lu Qin^1,2^, Yuanzhou He^1,2^, Jianping Zhao^1,2^, Yongjian Xu^1,2^, Xiansheng Liu^1,2^, Xiaochen Li^1,2^.

^1^Department of Pulmonary and Critical Care Medicine, Tongji Hospital, Tongji Medical College, Huazhong University of Science and Technology, Wuhan, China.

^2^Key Laboratory of Respiratory Diseases, National Ministry of Health of the People's Republic of China and National Clinical Research Center for Respiratory Disease, Wuhan, China.

^3^Department of Pediatrics, Tongji Hospital, Tongji Medical College, Huazhong University of Science and Technology, Wuhan, China.

**Running title:** Notch4 contributes to pulmonary hypertension

**Correspondence to:** MD. Xiaochen Li, Tongji Hospital, 1095 Jiefang Avenue, Wuhan 430030, China.

E-mail: lixiaochen3n2b@163.com.

**Supplementary Figure**

**
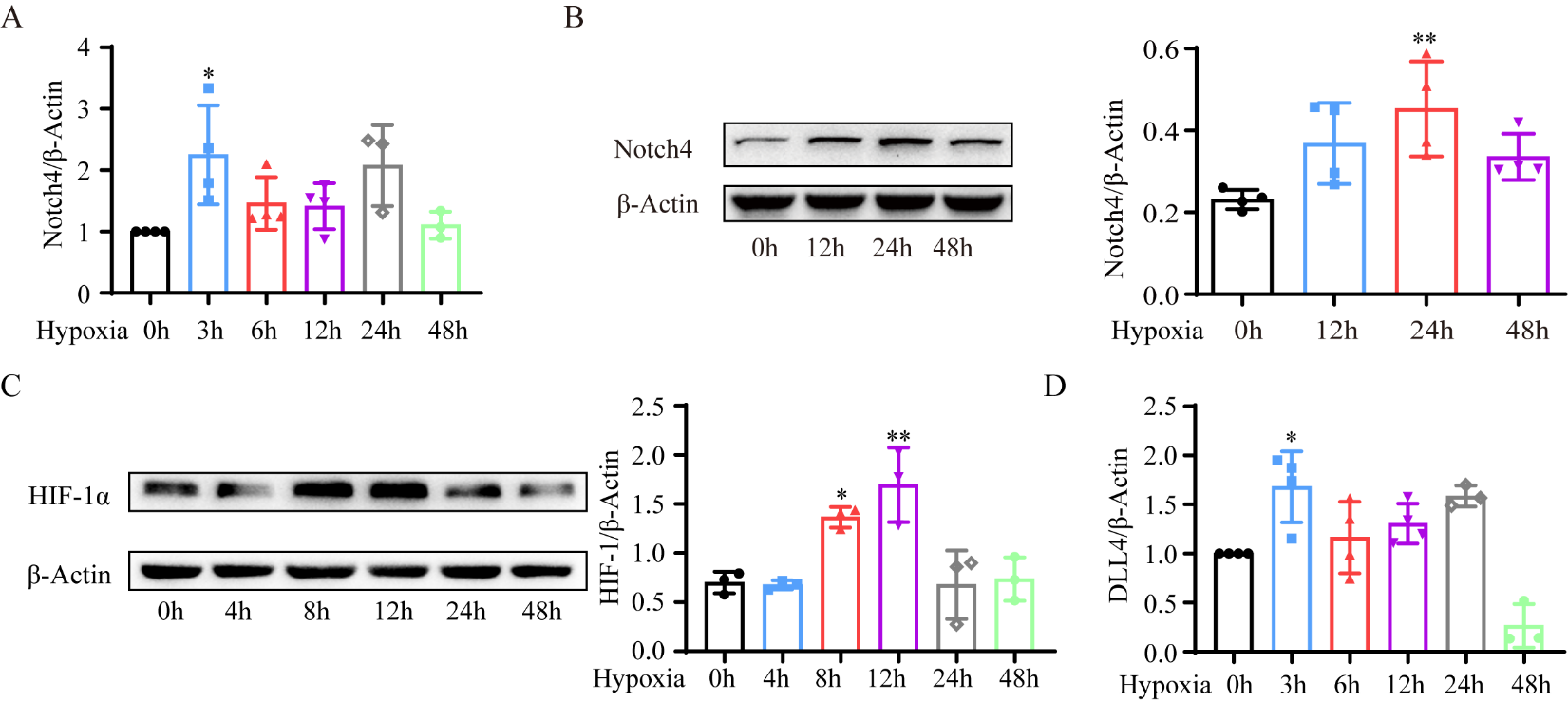
**

**Figure S1. Protein expressions in HPASMCs exposed to hypoxia.**

**(A)** The mRNA level of Notch4 in HPASMCs exposed to hypoxia for indicate time. **(B)** Protein expression of Notch4 in HPASMCs exposed to hypoxia for different time. **(C)** Protein expression of HIF-1α in HPASMCs exposed to hypoxia for different times. **(D)** The mRNA levels of DLL4. Data were presented as means ± SD. ^*^*P* < 0.05, ^**^*P*<0.01. One-way ANOVA followed by Tukey’s correction for post-hoc comparisons were performed for **A**-**F**.

**Supplementary Figure**

**
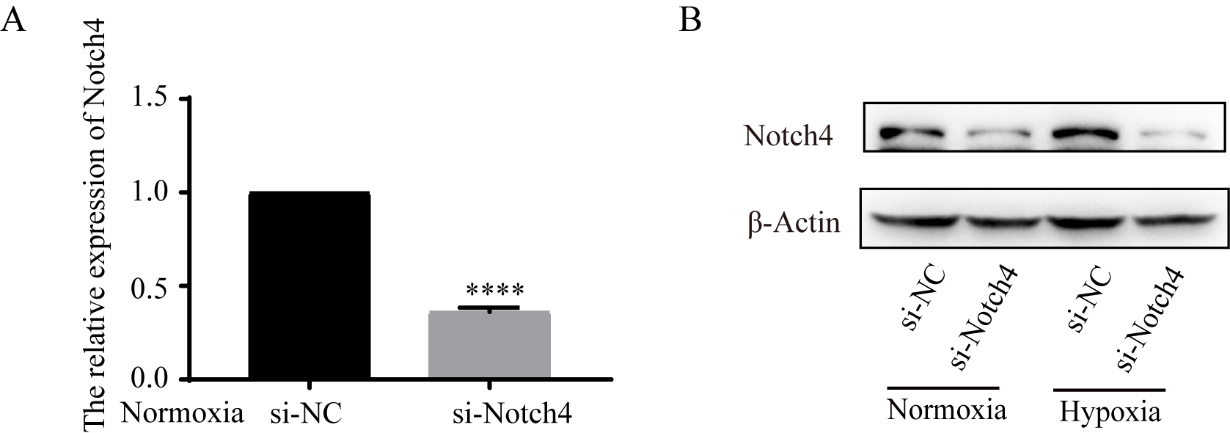
**

**Figure S2. Notch4 expression was knocked down in HPASMCs transfected with siRNA against Notch4.**

**(A)** The mRNA level of Notch4 in HPASMCs transfected with siRNA against Notch4 or negative control. **(B)** Protein expression of Notch4 in HPASMCs transfected with siRNA against Notch4 or negative control. Data were presented as means ± SD. ^****^*P* < 0.0001. Student’s t-test was performed for **A**. si-NC, negative control short interfering RNAs (siRNA); si-Notch4, the siRNA against Notch4.

**Supplementary Figure**

**
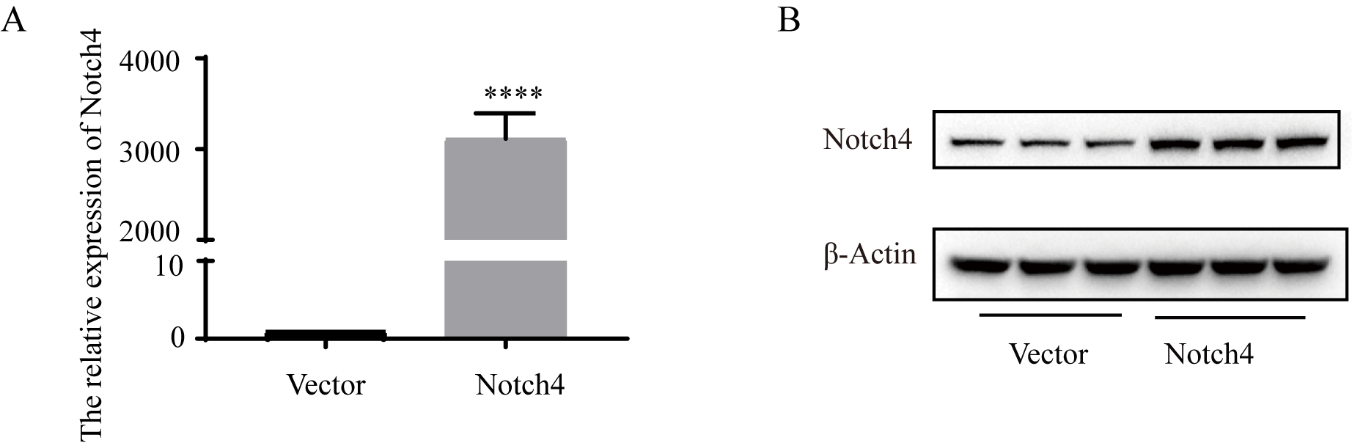
**

**Figure S3. Notch4 expression was overexpressed in HPASMCs transfected with Notch4 plasmid.**

**(A)** The mRNA level of Notch4 in HPASMCs transfected with Notch4 overexpression plasmid or negative control. **(B)** Protein expression of Notch4 in HPASMCs transfected with Notch4 overexpression plasmid or negative control. Data were presented as means ± SD. ^****^*P* < 0.0001. Student’s t-test was performed for **A**. Vector, negative control plasmid; Notch4, Notch4 plasmid.

**Supplementary Figure**


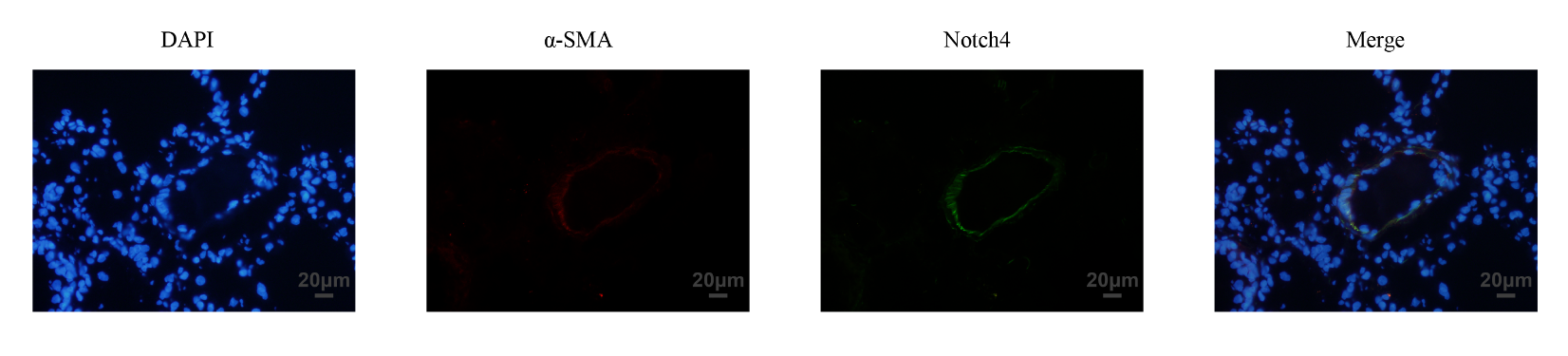


**Figure S4. Efficiency of intratracheal targeted delivery of Notch4 gene transfer to the lung vasculature.**

Immunofluorescence staining assay was performed to assess the efficacy of intratracheal targeted delivery of Notch4 gene transfer to the lung vasculature in AAVi-si-Notch4 therapeutic group. Magnification, x400; Bar, 20μm. α-SMA, α-smooth muscle actin.

**Major Resources Table**

**Animals (in vivo studies)**

| **Species** | **Vendor or Source** | **Background Strain** | **Sex** | **Persistent ID / URL** |
| --- | --- | --- | --- | --- |
| rat | Tongji Hospital | Sprague-Dawley | male |  |

**Cultured Cells**

| **Name** | **Vendor or Source** | **Sex** | **Persistent ID / URL** |
| --- | --- | --- | --- |
| Human Primary Artery Smooth Muscle Cell | Procell Life  Science & Technology | Unknown |  |

**Antibodies**

| **Targeted antigen** | **Vendor or Source** | **Catalog #** | **Persistent ID / URL** |
| --- | --- | --- | --- |
| PCNA | Proteintech | 10205-2-AP | https://www.ptglab.com/products/PCNA-Antibody-10205-2-AP.htm |
| Bax | Proteintech | 50599-2-Ig | https://www.ptglab.com/products/BAX-Antibody-50599-2-Ig.htm |
| Survivin | Proteintech | 10508-1-AP | https://www.ptglab.com/products/SURVIVIN-Antibody-10508-1-AP.htm |
| MMP9 | Proteintech | 10375-2-AP | https://www.ptglab.com/products/MMP9-Antibody-10375-2-AP.htm |
| MMP2 | Proteintech | 10373-2-AP | https://www.ptglab.com/products/MMP2-Antibody-10373-2-AP.htm |
| Bcl-2 | Boster | BM0200 | http://www.boster.com.cn/product/anti-bcl2-antibody-monoclonal-bcl-2-100_bm0200.html |
| α-SMA | Boster | BM0002 | http://www.boster.com.cn/product/anti-smooth-muscle-actin-a-sma-antibody-monoclonal-1a4_bm0002.html |
| Notch4 | Santa Cruz Biotechnology | sc-393893 | https://www.scbt.com/zh/p/notch-4-antibody-a-12?requestFrom=search |
| p-ERK | Cell Signaling  Technology | #4370 | https://www.cellsignal.cn/products/primary-antibodies/phospho-p44-42-mapk-erk1-2-thr202-tyr204-d13-14-4e-xp-rabbit-mab/4370?site-search-type=Products&N=4294956287&Ntt=p-erk&fromPage=plp |
| ERK | Cell Signaling Technology | #4695 | https://www.cellsignal.cn/products/primary-antibodies/p44-42-mapk-erk1-2-137f5-rabbit-mab/4695?site-search-type=Products&N=4294956287&Ntt=erk&fromPage=plp |
| p-JNK | Cell Signaling Technology | #4668 | https://www.cellsignal.cn/products/primary-antibodies/phospho-sapk-jnk-thr183-tyr185-81e11-rabbit-mab/4668?site-search-type=Products&N=4294956287&Ntt=%234668&fromPage=plp&_requestid=1056871 |
| JNK | Cell Signaling Technology | #9252 | https://www.cellsignal.cn/products/primary-antibodies/sapk-jnk-antibody/9252?site-search-type=Products&N=4294956287&Ntt=%239252&fromPage=plp&_requestid=1057016 |
| p-P38 | Cell Signaling Technology | #4511 | https://www.cellsignal.cn/products/primary-antibodies/phospho-p38-mapk-thr180-tyr182-d3f9-xp-rabbit-mab/4511?site-search-type=Products&N=4294956287&Ntt=%234511&fromPage=plp&_requestid=1057078 |
| P38 | Cell Signaling Technology | #8690 | https://www.cellsignal.cn/products/primary-antibodies/p38-mapk-d13e1-xp-rabbit-mab/8690?site-search-type=Products&N=4294956287&Ntt=%238690&fromPage=plp&_requestid=1057134 |

**Other**

| **Description** | **Description** | **Catalogue number** |
| --- | --- | --- |
| U0126 | MedChemExpress | HY-12031A |
| SP600125 | MedChemExpress | HY-12041 |
| SB203580 | MedChemExpress | HY-10256 |
| Cell Counting Kit-8 | Biosharp | BS350B |
| Edu Assay Kit | RiboBio | Cat #C10310-1 |
| Annexin V-FITC/PI Kit | KeyGEN BioTECH | Cat #KGA108 |
